# The Lactate–Lactylation Axis as a Metabolic–Epigenetic Framework for Therapeutic Adaptation in Esophageal Squamous Cell Carcinoma

**DOI:** 10.3390/cancers18182991

**Published:** 2026-09-16

**Authors:** Jingjie Yu, Yiyuan Cui, Sicong Li, Xinyu Guo, Nuo Li, Yuanye Gu, Yue Jin, Yufan Chen, Xinyu Li, Yijing Yan, Zhibin Wu, Chenxin Zhu, Li Feng

**Affiliations:** 1 Department of Oncology and Hematology, Dongzhimen Hospital, Beijing University of Chinese Medicine, Beijing 100700, China; 2 National Cancer Center/National Clinical Research Center for Cancer/Cancer Hospital, Chinese Academy for Medical Sciences and Peking Union Medical College, Beijing 100021, China; 3 Fangshan Hospital, Beijing University of Chinese Medicine, Beijing 102400, China; 4 Longhua Hospital, Shanghai University of Traditional Chinese Medicine, Shanghai 200032, China

**Keywords:** esophageal squamous cell carcinoma, lactate metabolism, lysine lactylation, metabolic reprogramming, therapeutic adaptation

## Abstract

Therapeutic failure in esophageal squamous cell carcinoma (ESCC) can arise as tumor cells and their microenvironment adapt to sustained treatment pressure. The lactate–lactylation axis may connect altered metabolism with adaptive treatment responses through changes in protein and chromatin regulation. However, how this axis contributes to therapeutic adaptation in ESCC and how its mechanistic evidence should be interpreted across different biological contexts remain incompletely defined. Here, we review lactate sources, the regulatory landscape of lysine lactylation, and emerging evidence linking this axis to heterogeneous treatment responses in ESCC. Studies in ESCC implicate several lactylation-related processes in therapeutic adaptation. These include poly(ADP-ribose) polymerase 1 (PARP1) K654 lactylation in DNA damage responses, signal transducer and activator of transcription 3 (STAT3) K631 and nudix hydrolase 21 (NUDT21) K23 lactylation in programmed cell death susceptibility, and the NIPA-like domain containing 1 (NIPAL1)–histone H3 lysine 18 lactylation(H3K18la) and hypoxia-inducible factor 1α (HIF-1α) K172la axes in immune microenvironment remodeling. The strength of evidence differs across individual mechanisms. Several site-specific lactylation events have been identified in ESCC models or patient-derived specimens and supported by functional validation, whereas the role of lactylation-associated pathways in predicting immunotherapy response requires further clinical investigation. Therefore, the lactate–lactylation axis is best viewed as an emerging metabolic–epigenetic network that connects cellular metabolic states with adaptive treatment phenotypes rather than as a universal resistance mechanism. Integrating longitudinal clinical cohorts, multi-omics profiling, and mechanistic studies will be critical for defining how lactate–lactylation signatures may support patient stratification and precision therapeutic strategies in ESCC.

## Simple Summary

Esophageal squamous cell carcinoma is an aggressive cancer in which treatment failure can reflect adaptation by tumor cells and the surrounding immune environment to chemotherapy and immunotherapy. Lactate, traditionally viewed as a glycolytic byproduct, can also influence gene expression and protein function through lysine lactylation. This review examines how the lactate–lactylation axis may contribute to therapeutic adaptation in esophageal squamous cell carcinoma. We distinguish evidence linking lactate metabolism to treatment outcomes from evidence showing lactylation-associated changes and from stronger studies that directly test a defined lactylation site. Specific lactylation events have been linked to DNA repair, regulated cell death, and immune evasion, although clinical validation remains limited. The review highlights the need for reliable site-specific measurements and prospective patient studies before these mechanisms can guide treatment selection or new therapies.

## 1. Introduction

Esophageal squamous cell carcinoma (ESCC) accounts for the majority of esophageal cancers in East Asia. Treatment has shifted from a primarily local, surgery-oriented approach toward multidisciplinary management combining neoadjuvant therapy, surgery, and systemic treatment. Randomized trials have established neoadjuvant chemoradiotherapy (nCRT) followed by surgery as a standard strategy for locally advanced ESCC, with long-term analyses supporting its survival advantage [1,2,3,4]. Immune checkpoint inhibitors (ICIs) have improved outcomes in advanced disease, yet many patients receiving first- or second-line treatment do not achieve durable responses [5,6,7,8,9]. Conventional clinical factors, including tumor stage, treatment selection, and programmed death-ligand 1 (PD-L1) expression, do not fully account for the substantial variation in therapeutic responses among individuals [10,11,12,13]. These observations point to adaptive states that may emerge under sustained treatment pressure.

Such heterogeneity cannot be explained solely by differences in baseline drug sensitivity. Anticancer therapies impose distinct forms of stress, including DNA damage, oxidative injury, and immune-mediated selection. Tumor cells can respond by strengthening repair pathways, altering cell death thresholds, and reshaping their metabolic programs. Immunotherapy introduces additional selective forces through interactions between malignant cells and immune populations within the tumor microenvironment. Identifying regulatory mechanisms shared across these stress responses may therefore provide insight into why some tumors persist despite multimodal treatment.

Metabolic reprogramming is increasingly recognized as an important component of tumor adaptation. The Warburg effect describes the preference of cancer cells for aerobic glycolysis even under normoxic conditions, resulting in enhanced lactate production and accumulation [14]. Beyond glycolysis, lactate can contribute to signaling and epigenetic regulation that influences tumor progression, microenvironmental remodeling, and therapeutic responses [15,16,17]. Public dataset analyses have linked lactate-associated gene signatures with prognosis and immune cell composition in ESCC, suggesting a potential relationship between lactate metabolism and treatment heterogeneity [18]. Other metabolic and functional studies have shown that lactate dehydrogenase A (LDHA)-driven glycolytic remodeling contributes to ESCC treatment resistance, while changes in hypoxia signaling, mitochondrial activity, and carbon metabolism are associated with chemoradiotherapy sensitivity [19,20,21,22]. However, most previous work has focused on lactate production and metabolic flux, leaving the mechanisms through which lactate-derived signals are converted into adaptive cellular programs incompletely characterized.

A key advance in this field came in 2019, when Zhang and colleagues identified lysine lactylation (Kla) as a histone post-translational modification, providing a direct connection between cellular metabolism and transcriptional regulation [23,24]. Subsequent studies have shown that lactylation occurs not only on histones but also on diverse non-histone proteins, influencing processes such as DNA repair, cell death regulation, and immune signaling [25,26,27,28]. Thus, lactate accumulated during treatment may act as a signaling input through lactylation, although the functional consequences depend on the modified substrate.

**Figure 1:**
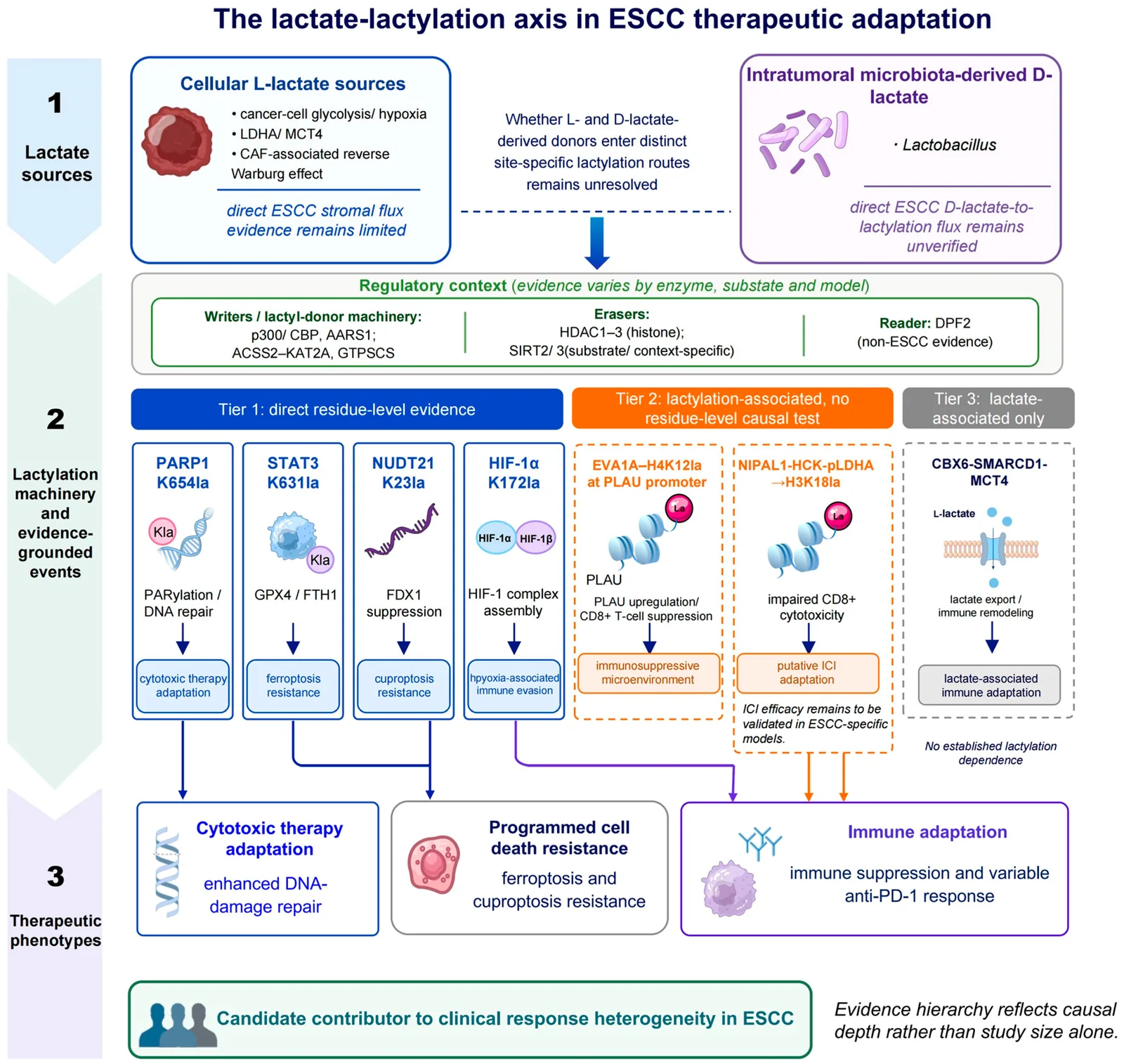
The lactate–lactylation axis as a metabolic–epigenetic framework for therapeutic adaptation in esophageal squamous cell carcinoma (ESCC). The framework is organized into three layers. (**1**) Lactate sources: intratumoral L-lactate arises from cancer-cell glycolysis and hypoxia-driven LDHA/MCT4 activity, with cancer-associated fibroblasts (CAFs) considered a plausible additional contributor. Intratumoral Lactobacillus can generate D-lactate. Whether L- and D-lactate enter distinct site-specific lactylation pathways remains unresolved. (**2**) Regulatory machinery: lactylation involves p300/CBP and AARS1 catalytic activity, together with ACSS2–KAT2A- and GTPSCS-associated lactyl-donor pathways, and is reversed by erasers (HDAC1–3 and SIRT2/3) with substrate- and context-specific activity; DPF2 serves as a reader in non-ESCC systems. (**3**) Evidence hierarchy and adaptive phenotypes: Tier 1 (blue) comprises ESCC-specific events with direct residue-level functional evidence—PARP1 K654 lactylation enhancing PARylation and DNA repair to support cytotoxic therapy adaptation; STAT3 K631 lactylation upregulating GPX4/FTH1 to confer ferroptosis resistance; NUDT21 K23 lactylation suppressing FDX1 to promote cuproptosis resistance; and HIF-1α K172 lactylation driving HIF-1 complex assembly and hypoxia-associated immune evasion. Tier 2 (orange) comprises lactylation-associated pathways supported by functional studies: the EVA1A–H4K12la–PLAU axis shaping an immunosuppressive microenvironment, and the NIPAL1–HCK–p-LDHA–H3K18la circuit impairing CD8^+^ T-cell cytotoxicity as a putative mechanism of immune checkpoint inhibitor (ICI) adaptation. Tier 3 (grey) comprises lactate-associated metabolic remodeling (CBX6–SMARCD1–MCT4-mediated lactate efflux) linked to immune modulation. Arrows connect each event to its corresponding therapeutic phenotype: cytotoxic therapy adaptation, programmed cell death resistance, or immune adaptation. The evidence hierarchy reflects causal depth and mechanistic specificity rather than study size or clinical maturity. Supporting studies are detailed in Table 1 and Table S1. Abbreviations: CAF, cancer-associated fibroblast; ICI, immune checkpoint inhibitor; Kla, lysine lactylation; LDHA, lactate dehydrogenase A; MCT4, monocarboxylate transporter 4; PARylation, poly(ADP-ribosyl)ation. Figure created with scifig.com and SciFig v1.5.0 (VSF); Smart Servier (CC BY 4.0); NIH (public domain). Jingjie Yu. (2026).

Recent reviews have summarized lactylation in cancer broadly and in esophageal cancer [29,30]. However, these reviews principally organize the literature around tumor progression, hypoxia-driven metabolic reprogramming, immune escape, and candidate therapeutic targets. They do not explicitly distinguish whether a treatment-related phenotype is supported by lactate/metabolic evidence, lactylation-associated evidence, or mechanistically validated site-specific lactylation evidence. This review therefore focuses specifically on therapeutic adaptation in ESCC. We examine DNA damage responses, programmed cell death regulation, and immune microenvironment remodeling through an evidence hierarchy that weighs residue-level causality, functional validation, and treatment relevance rather than study size alone. This framework distinguishes biological plausibility from site-specific causal evidence and identifies the translational gaps that must be resolved before the lactate–lactylation axis can inform patient stratification or precision intervention (Figure 1).

**Table 1:** Evidence hierarchy for lactylation-related therapeutic adaptation in ESCC.

Reference	Tier	Therapeutic Context	Lactate Source & Metabolic Driver	Site-Specific Lactylation	Validation Type	Functional Consequence	Adaptive Phenotype	Key Limitation
Peng et al. [31]	1	Neoadjuvant chemotherapy	Chemotherapy-associated L-lactate accumulation	PARP1 K654la	Lactylome LC-MS/MS; IP-WB; K654R; genetic-code expansion; SIRT2 perturbation; drug-response assays	Enhances PARylation and DNA damage repair	Chemotherapy adaptation/resistance	Single-center cohort; no independent biomarker validation
Wang et al. [32]	1	Ferroptosis pressure	Intratumoral Lactobacillus-derived D-lactate	STAT3 K631la	Bacterial metabolomics; LC-MS/MS; K631R; *ldhD* knockout; ferroptosis assays; xenografts	Promotes STAT3 activity and GPX4/FTH1 expression	Ferroptosis resistance	No patient treatment-response endpoint; D-lactate donor chemistry requires further clarification
Lin et al. [33]	1	Copper-ionophore-induced cuproptosis	LDHA-associated L-lactate production	NUDT21 K23la	LC-MS/MS; IP-WB; K23R; AARS1/HDAC2 perturbation; protein-interaction assays; rescue; xenografts	Promotes CFIm assembly and *FDX1* 3′ UTR lengthening	Cuproptosis resistance	No treatment-response cohort; biomarker threshold and compatibility with standard therapy remain unknown
Cong et al. [34]	1	Anti-PD-1 immune checkpoint blockade	Hypoxia-associated glycolysis and lactate accumulation	HIF-1α K172la	LC-MS/MS; K172R; co-IP; LDHA/lactate perturbation and rescue; CD8+ assays; immunocompetent allografts	Promotes HIF-1 complex assembly and immunosuppressive transcription	Immune evasion and reduced anti-PD-1 sensitivity	Predominantly preclinical; no patient ICB-response cohort
Yuan et al. [35]	2	Immune evasion	EVA1A-associated glycolytic lactate accumulation	H4K12la enrichment at the *PLAU* promoter	EVA1A perturbation; H4K12la immunoblotting; ChIP-qPCR; PLAU rescue; CD8+ co-culture; PBMC-reconstituted xenografts	Promotes PLAU expression and suppresses CD8+ T-cell activity	Immunosuppressive microenvironment	No direct residue-level perturbation, writer/eraser test, or ICB-response model
Chen et al. [36]	2	Anti-PD-1 immune checkpoint blockade	NIPAL1-HCK-p-LDHA-driven glycolytic flux	H3K18la at the *NIPAL1* promoter	Patient cohorts; co-IP/PLA; LDHA Y10F rescue; H3K18la ChIP-qPCR; CD8+ assays; MC38 anti-PD-1 model	Reinforces NIPAL1-HCK-p-LDHA-H3K18la signaling and impairs CD8+ cytotoxicity	Immune evasion and ICB-associated resistance	p300 inhibition is not lactylation-specific; efficacy was tested in MC38, not ESCC, allografts
Wang et al. [37]	3	Immune remodeling	CBX6-SMARCD1-MCT4-mediated lactate efflux	Not established	*Cbx6/Smarcd1* perturbation; ChIP/CUT&Tag; CD8+ co-culture; MCT inhibition; mouse models	Remodels CCL8 and MCT4 expression with CD8+ T-cell exhaustion	Lactate-associated immune remodeling	Lactylation dependence and a site-specific mechanism remain unestablished

Evidence-tier definitions. Tier 1: ESCC-specific evidence linking a defined lactylation site to a therapy-relevant phenotype or biological mechanism, supported by direct site-level functional validation (e.g., site-directed mutagenesis or an equivalent causal perturbation) and mechanistic elucidation in vitro and/or in vivo. Writer/eraser manipulation and orthogonal analytical validation may provide complementary support but are not, by themselves, sufficient for Tier 1 assignment. The mechanism must be validated at the specific lactylated residue, rather than only at the global lactylation or protein level. Patient-derived evidence may include treatment-response cohorts, prospective biopsies, or well-annotated archival ESCC tissues demonstrating clinically relevant biological associations. Single-center studies with robust site-specific causal validation may qualify as Tier 1; multicenter or prospective validation strengthens external and clinical validity but is not required for tier assignment. Tier 2: ESCC evidence supporting lactylation-dependent regulation of a therapy-relevant phenotype, but lacking direct functional manipulation of the specific lactylated residue. Evidence may include global or histone-level lactylation modulation (e.g., writer/eraser pharmacological inhibition, pan-Kla antibody detection), upstream mechanistic perturbation (e.g., LDHA Y10F affecting lactate production but not the lactylation site itself), patient associative data without site-specific validation, or preclinical functional models without patient tissue confirmation. Tier 3: Lactate-associated therapeutic-ecosystem remodeling in ESCC without established lactylation dependence, or mechanistic evidence extrapolated from non-ESCC systems. All abbreviations used in this table are defined in the Abbreviations section.

Literature search and selection. PubMed was searched from database inception through August 2026 using combinations of “esophageal squamous cell carcinoma,” “ESCC,” or “esophageal cancer” with “lactate,” “lactic acid,” “glycolysis,” “LDHA,” “MCT4,” “lactylation,” “lysine lactylation,” or “Kla,” and terms for chemotherapy, cisplatin, 5-fluorouracil, radiotherapy, immunotherapy, treatment response, or resistance. We prioritized original ESCC studies that used patient-derived material or functional cell or animal models to examine treatment-relevant phenotypes. Duplicate reports, studies unrelated to ESCC or the review question, and claims unsupported by the reported experiments were excluded from the disease-specific synthesis. Non-ESCC studies were selected only to explain biochemical mechanisms, analytical methods, or intervention-related limitations not yet established in ESCC; these studies were identified as contextual evidence and did not determine ESCC-specific evidence-tier assignments. PubMed was the primary database because it covers the biomedical literature relevant to this review. We also traced citations in relevant primary studies and reviews to identify additional reports.

## 2. Foundations of the Lactate–Lactylation Axis in ESCC: Lactate Sources and Regulatory Contexts

Lactate accumulation in ESCC is unlikely to arise from a single metabolic process. At least two distinct sources are recognized: L-lactate produced by tumor cell glycolysis and D-lactate generated by intratumoral microbial metabolism. Lactate exchange involving tumor cells, stromal components, and immune populations may also influence the local metabolic environment, although direct evidence for these interactions in ESCC remains scarce. Current studies have mainly focused on tumor-derived L-lactate and microbiota-associated D-lactate. These two lactate enantiomers differ in their stereochemical properties, metabolic generation and clearance pathways, and potentially their downstream regulatory consequences. Consequently, total lactate measurements alone may provide limited information about the origin of lactate accumulation or the functional consequences associated with different lactate species [38].

### 2.1. Tumor Cell-Derived L-Lactate

Tumor cell-derived L-lactate is primarily generated through enhanced glycolytic activity driven by hypoxia and oncogenic signaling. Hypoxia-inducible factor 1α (HIF-1α) promotes glucose uptake and glycolytic flux by transcriptionally upregulating glucose transporter 1 (GLUT1) and hexokinase 2 (HK2). The resulting pyruvate is subsequently converted into L-lactate by lactate dehydrogenase A (LDHA), which is then exported into the tumor microenvironment through monocarboxylate transporter 4 (MCT4). In ESCC, this glycolytic phenotype has been repeatedly reported and is associated with malignant progression and reduced treatment sensitivity [39,40].

In ESCC, lactate may have regulatory roles beyond being a glycolytic end product. Once accumulated, lactate can participate in metabolic–epigenetic regulation through lactylation of metabolic regulatory proteins, including Axin1, SHMT2, and DCBLD1, potentially establishing a reinforcing feedback loop that sustains glycolytic dependency and adaptive metabolic phenotypes [41,42,43].

Cancer-associated fibroblasts (CAFs) may represent an additional local source of L-lactate in the ESCC microenvironment. In the reverse Warburg model, glycolytically reprogrammed CAFs export lactate, commonly through MCT4, which can be imported by adjacent tumor cells through MCT1 to support oxidative metabolism and signaling [44,45]. Although this stromal–epithelial lactate shuttle has been described in several solid tumors, direct spatial or flux-resolved evidence that CAF-derived lactate drives lactylation or therapeutic adaptation in ESCC remains lacking. CAFs should therefore be considered a plausible, but not yet validated, contributor to local lactate availability in ESCC.

### 2.2. Intratumoral Microbiota-Derived D-Lactate

The intratumoral microbiota has emerged as an important component of the gastrointestinal tumor ecosystem [46]. ESCC develops in direct contact with the external environment and is continuously exposed to microorganisms from the oral cavity and dietary sources. This anatomical context creates a distinct metabolic setting in which bacteria residing within tumors may contribute to local D-lactate production. In addition to Lactobacillus, other genera present or enriched in ESCC, including Streptococcus and Leptotrichia, may influence the local metabolic milieu and contribute to the local lactate pool.

Multi-region analyses of ESCC tissues combining 16S rRNA sequencing, untargeted metabolomics, and transcriptomic profiling have suggested that Lactobacillus species may contribute to intratumoral D-lactate generation and are associated with unfavorable survival outcomes [32]. Evidence from other cancer models further indicates that microbial lactate can influence tumor-associated immune states, including promotion of an M2-like tumor-associated macrophage phenotype [47] and facilitation of metastatic colonization [48]. Whether these observations reflect similar biological processes in ESCC remains unclear, and the functional role of microbiota-derived D-lactate in this disease requires direct mechanistic validation.

### 2.3. Stereochemical and Enzymatic Considerations: L- and D-Lactate in Lactylation

L- and D-lactyllysine are stereochemically distinct modifications and may also differ in analytical detectability [49]. Notably, anti-L-lactyllysine antibodies can display strong stereoselectivity [50]. Whether mammalian lactylation machinery distinguishes L- from D-lactate stereoisomers remains unresolved. Host tumor cells predominantly generate L-lactate through LDHA-mediated pyruvate reduction, whereas intratumoral microbiota may contribute additional lactate species to the local metabolic pool [51]. In ESCC, Lactobacillus-derived D-lactate has been linked to STAT3 K631 lactylation [32]. Other genera reported in the ESCC-associated microbiota, including Streptococcus and Leptotrichia, may also contribute to the local metabolic milieu, although their quantitative contribution to intratumoral lactate—and particularly to D-lactate—has not been established [52,53]. The stereochemical composition of the lactate produced by these organisms in tumors remains insufficiently defined. Whether established mammalian lactylation regulators such as p300/CBP, AARS1, ACSS2, or GTPSCS exhibit stereochemical preference for L- versus D-lactate or their activated lactyl donors has not been systematically established. This uncertainty is particularly relevant because p300/CBP regulates multiple lysine acylations, so pharmacological perturbation of p300 cannot be interpreted as lactylation-specific evidence.

The metabolic handling of D-lactate adds further complexity. In ESCC stem-like cells, the CDK7–YAP–LDHD axis promotes mitochondrial D-lactate catabolism, resulting in D-lactate depletion and pyruvate accumulation, while protecting these cells from D-lactate-induced ferroptosis and supporting stem-like properties [54]. However, how this metabolism contributes to protein lactylation remains unresolved. Conceptually, D-lactate could undergo metabolic recycling before entering a canonical L-lactate-derived lactylation pathway, or alternatively contribute through a distinct D-lactyl donor or noncanonical intermediate. At present, these possibilities remain hypothetical, and direct biochemical evidence distinguishing host enzymatic recycling from stereospecific D-lactylation is lacking.

Delactylases may also distinguish lactylation stereoisomers. Biochemical studies have established HDAC1–3 as delactylases and demonstrated stereochemical preferences, with class I HDACs generally removing K(D-la) more efficiently than K(L-la), while HDAC3 shows the least stereochemical discrimination [50]. However, the extent to which individual HDACs or sirtuins display substrate- and context-dependent preferences for L- versus D-lactyllysine in ESCC remains incompletely defined. These uncertainties are particularly important for interpreting the Lactobacillus–D-lactate–STAT3 K631la pathway: although D-lactate dependence and the functional requirement of STAT3 K631 have been demonstrated [32], the biochemical route connecting extracellular D-lactate to installation of the K631 lactyl mark has not yet been resolved.

### 2.4. Crosstalk Between Lysine Lactylation and Acetylation at Shared Residues

A further layer of complexity is the potential crosstalk between lysine lactylation and acetylation. These modifications share regulators, including p300/CBP and several HDACs, and can occur at the same lysine site. They are mutually exclusive at that residue on a given molecule, although both may occur on different molecules or at other residues. H3K18 is a representative shared modification site, but its relative acetylation versus lactylation is likely determined by a combination of acyl-donor availability, writer substrate preference, chromatin context, and eraser activity rather than by the ratio of acetyl-coenzyme A (acetyl-CoA) to lactyl-coenzyme A (lactyl-CoA) alone. Reviews of the emerging lactylation field further emphasize that extensive overlap between acetylated and lactylated proteins complicates modification-specific interpretation [55].

Metabolic state may nevertheless bias this balance. ACSS2 and GTPSCS can generate lactyl-CoA and cooperate with KAT2A or p300 to support histone lactylation, whereas p300/CBP remains a broad acyltransferase rather than a lactylation-specific writer [56]. Thus, increased glycolytic flux and lactate availability may create conditions more permissive for lactylation, but this should not be viewed as a simple substrate-replacement switch. Notably, lactate itself can also serve as a carbon source for histone acetylation through nuclear LDH-dependent metabolism, indicating that lactate may feed both lactylation and acetylation pathways depending on intracellular metabolic routing [57].

For non-histone proteins, analogous competition remains largely unresolved. PARP1 contains established p300/CBP-dependent acetylation sites, whereas K654 lactylation promotes PARylation and DNA repair in ESCC; however, direct acetylation–lactylation competition at K654 has not been demonstrated. Therefore, p300 or HDAC perturbation in ESCC should be interpreted cautiously because these interventions may simultaneously affect multiple lysine acylation states.

### 2.5. Analytical Considerations for Detecting and Validating Lactylation

Analytical confidence is central to the interpretation of lactylation-dependent mechanisms. Discovery studies commonly use affinity enrichment followed by high-resolution LC-MS/MS. Detecting a candidate lactylated peptide alone does not establish its modification identity or precise residue location. Site-localization confidence, fragmentation quality, and discrimination from related lysine acylations should therefore be considered. Antibody-based assays are useful for screening and tissue profiling, but pan-Kla and site-specific antibodies require validation against unmodified and chemically related modified peptides because antibody recognition can be modification- and stereoisomer-selective [23,50].

Quantitative lactylome measurements generally provide relative signals, not direct estimates of site occupancy. Antibody enrichment, ionization efficiency, and concurrent changes in protein abundance can affect apparent lactylation differences [31]. Candidate sites proposed as biomarkers should therefore be supported by orthogonal validation, ideally including MS/MS confirmation, synthetic modified peptide standards where feasible, site-directed mutagenesis or genetic-code expansion, regulatory perturbation, and functional rescue. This requirement is particularly important for ESCC studies that nominate site-specific lactylation events as treatment-response biomarkers [58].

### 2.6. Molecular Basis of Lactylation Regulation

Lactylation is a lactate-derived post-translational modification (PTM) that links cellular metabolism with epigenetic and protein-level regulation [58,59]. Its regulation is not mediated by a single writer–eraser pathway, but depends on substrate availability, enzyme context, and the modified protein.

Evidence for proposed lactylation writers should be distinguished by mechanism. p300/CBP can catalyze histone lactylation [23]. The ACSS2–KAT2A axis and GTPSCS can support lactyl-CoA generation and lactylation in defined settings [56,60]. By contrast, AARS1 has been reported to display direct lactyltransferase activity toward specific non-histone substrates [61]. These mechanisms differ in biochemical support, substrate specificity, and disease context.

Evidence for delactylases is likewise substrate- and context-dependent. HDAC1–3 have been characterized as histone delactylases, whereas SIRT3-mediated removal of H3K9 lactylation has been reported in esophageal cancer [50,62]. In ESCC, the most direct evidence is site-specific: SIRT2 regulates PARP1 K654la, while AARS1 and HDAC2 oppositely regulate NUDT21 K23la [31,33]. p300-dependent H3K18la has also been implicated in the NIPAL1-associated immune-evasion circuit, although p300 inhibition cannot be interpreted as lactylation-specific because p300/CBP regulates multiple acylation and transcriptional programs [36].

Reader evidence remains more limited in ESCC. Double PHD fingers 2 (DPF2) recognizes histone lactylation in other biological systems [63], but its substrate specificity and functional relevance in ESCC require direct validation. Therefore, each proposed writer, eraser, or reader should be interpreted according to its biochemical support, substrate context, and ESCC-specific evidence.

Lactylation occurs on both histone and non-histone substrates. Histone modifications, including H3K18la and histone H3 lysine 9 lactylation (H3K9la), can influence transcriptional states, whereas non-histone lactylation may regulate DNA repair, cell death, metabolism, and immune signaling. Sustained lactylation has been associated with metabolic and epigenetic remodeling in immune cells, although its relevance to ESCC remains unclear [64,65].

## 3. Disease-Relevant and Therapeutic Evidence of the Lactate–Lactylation Axis in ESCC

Throughout this section, we distinguish three related but non-equivalent evidence categories. Lactate/metabolic evidence links lactate production, transport, glycolytic flux, hypoxia, LDHA, or MCT activity to therapy-relevant phenotypes without establishing lactylation dependence. Lactylation-associated evidence links global or site-associated lactylation signals to a phenotype but lacks a residue-level causal test. Mechanistically validated site-specific lactylation evidence requires identification of a defined lactylated residue, direct perturbation of that residue, and demonstration that the modification alters the relevant phenotype. These categories inform, but do not replace, the Tier 1–3 hierarchy, which reflects causal depth and treatment relevance rather than cohort size alone.

### 3.1. Human Therapeutic Evidence: Lactylation and Metabolic Remodeling Under Chemotherapy Pressure

A neoadjuvant chemotherapy (NAC) study provides direct human-based evidence linking lactylation to treatment adaptation in ESCC. Proteomic and lactylomic profiling of 62 tumor and matched adjacent tissues from 31 patients, including NAC-treated and untreated cases, revealed extensive lactylation changes after chemotherapy, with a predominant effect on non-histone proteins. At the mechanistic level, DNA damage and increased lactate availability promoted poly(ADP-ribose) polymerase 1 (PARP1) K654 lactylation, enhancing poly(ADP-ribosyl)ation (PARylation) activity and DNA damage repair capacity [31]. Independent prospective cohorts are needed to determine whether PARP1 K654 lactylation predicts therapeutic response.

Another site-specific event, the D-lactate–signal transducer and activator of transcription 3 (STAT3) K631 lactylation axis, has been linked to increased glutathione peroxidase 4 (GPX4) and ferritin heavy chain 1 (FTH1) expression through multi-omics analysis and functional experiments, and is associated with patient survival outcomes [32]. Its contribution to treatment adaptation remains to be clarified, as the study did not include chemotherapy-specific response assessments.

A broader body of work has examined lactate metabolism in ESCC treatment sensitivity, although these studies should be distinguished from lactylation-dependent mechanisms. For example, circular RNA *GOT1* (circGOT1) promotes glycolysis and decreases cisplatin sensitivity [66], while alterations in GLUT1, amplified in liver cancer 1 (ALC1), and chaperonin containing TCP1 subunit 4 (CCT4) affect glycolytic remodeling and chemotherapy responses [67,68,69]. Metabolic regulation mediated by sirtuin 1 (SIRT1) and nicotinamide N-methyltransferase (NNMT) has also been implicated in cisplatin and 5-fluorouracil sensitivity through changes in redox homeostasis [70,71].

Similar observations have been reported in radiotherapy settings. Manipulation of DiGeorge syndrome critical region gene 5 (*DGCR5*), HIF-1α, or TP53-induced glycolysis and apoptosis regulator (TIGAR), as well as dichloroacetate treatment, can influence ESCC radiosensitivity by altering hypoxia adaptation, glycolytic activity, or mitochondrial reactive oxygen species (ROS) production [72,73,74,75]. ^18^F-fluorodeoxyglucose positron emission tomography (FDG-PET) studies further suggest that changes in tumor glucose metabolism are associated with pathological response after chemoradiotherapy [76].

Taken together, current data support a relationship between lactate-associated metabolic remodeling and treatment response in ESCC, but direct evidence for site-specific lactylation remains uneven. Validated events span chemotherapy-associated DNA repair, cell death resistance, and immune evasion. Whether radiotherapy induces functionally relevant lactylation remains largely unknown. Clarifying this gap is needed to determine the contexts in which the lactate–lactylation axis contributes most strongly to therapeutic adaptation.

### 3.2. Lactylation-Associated Molecular Features and Disease Progression: Supportive Evidence

Beyond treatment-related studies, lactylation has also been implicated in ESCC progression. A lactylomic analysis comparing ESCC tissues with and without lymph node metastasis (LNM) identified a global increase in lactylation in metastatic tumors, with 91 lysine lactylation (Kla) sites showing significant upregulation. Among these alterations, enolase 1 (ENO1) lactylation was positively associated with the migratory capacity of ESCC cells, suggesting that lactylation remodeling contributes to metastatic behavior [77].

Functional studies have further examined how individual lactylation events influence ESCC biology. Hypoxia-induced histone H3K9 lactylation promotes laminin subunit gamma 2 (LAMC2) transcription [78], whereas histone H2B cluster member 9 (H2BC9) lactylation has been linked to ESCC progression through modulation of the Wnt/β-catenin pathway [79]. In another study, the long non-coding RNA AP001885.4 was shown to enhance ESCC cell proliferation through lactylation-associated histone regulation and nuclear factor-κB (NF-κB)-dependent transcriptional activation [80]. Together, these findings support a role for lactylation in shaping malignant phenotypes in ESCC and provide additional biological context for understanding how lactylation may influence treatment adaptation.

At the prognostic level, transcriptomic analyses have yielded lactate- or lactylation-related gene signatures for survival prediction and characterization of immune infiltration patterns in esophageal cancer [18,81,82,83]. These models may help generate hypotheses for molecular stratification. However, their clinical application requires further validation due to limited external validation, heterogeneous detection methods, and the lack of prospective evaluation.

### 3.3. Evidence Related to Immunotherapy: An Emerging Mechanistic Link

Recent ESCC studies have identified two lactylation-related pathways with potential relevance to immune checkpoint blockade: the NIPA-like domain containing 1 (NIPAL1)–hematopoietic cell kinase (HCK)–phosphorylated LDHA–histone H3 lysine 18 lactylation (H3K18la) axis and hypoxia-inducible factor 1α (HIF-1α) K172 lactylation [34,36]. The NIPAL1-related pathway is supported by patient-derived associations and functional studies using immunocompetent non-ESCC models, providing mechanistic support linking lactate metabolism, lactylation, and anti-programmed cell death-1 (anti-PD-1) response. HIF-1α K172 lactylation provides additional mechanistic insight by demonstrating how a specific lactylation event influences immune-related signaling and therapeutic sensitivity in preclinical settings. Despite these findings, neither mechanism has been validated in prospective cohorts designed to predict immunotherapy benefit. Thus, lactylation-mediated immune regulation represents an emerging mechanism in ESCC, although its value as a clinically predictive determinant of immune checkpoint inhibitor efficacy remains to be established. The supporting evidence and mechanistic details are discussed in Section 4.3.

## 4. Mechanistic Roles of the Lactate–Lactylation Axis in ESCC Therapeutic Adaptation

Therapeutic adaptation in ESCC arises from changes within tumor cells and interactions with the surrounding microenvironment. Persistent treatment exposure can induce or select tumor populations with enhanced DNA damage repair capacity, altered sensitivity to programmed cell death, and immunosuppressive features that support survival after therapy. Lactate metabolism and lactylation may contribute to these processes through downstream transcriptional and cellular responses. However, the strength of evidence differs across individual mechanisms. Some lactylation-dependent events have been directly demonstrated in ESCC models or patient-derived samples, whereas other pathways remain supported mainly by evidence from other malignancies. Their clinical relevance in ESCC treatment response therefore requires further investigation.

### 4.1. Lactylation-Enhanced DNA Damage Repair and Survival Adaptation Under Cytotoxic Stress

Chemotherapy and radiotherapy induce tumor cell killing through distinct forms of genotoxic stress. Chemotherapeutic agents such as cisplatin generate DNA adducts and replication-associated damage, whereas radiation primarily causes single- and double-strand DNA breaks. These insults activate the DNA damage response (DDR), which is a major determinant of whether tumor cells survive treatment. Following DNA damage recognition, PARP1 undergoes conformational activation and catalyzes poly(ADP-ribosyl)ation (PARylation), generating poly(ADP-ribose) chains that recruit downstream repair factors [84,85]. In PARP1, lysine 654 (K654) lies within the linker region connecting the WGR and helical domains (HD), a structural region involved in transmitting damage-sensing signals to the catalytic domain [86]. Evidence from ESCC directly implicates PARP1 lactylation in cytotoxic stress adaptation. Cisplatin exposure or lactate stimulation increases PARP1 K654 lactylation and PARylation activity, whereas mutation of K654 to arginine attenuates these effects. Sirtuin 2 (SIRT2)-mediated delactylation supports the reversibility of this modification. Together with lactylomic changes identified in ESCC patient samples after neoadjuvant therapy, these findings support a mechanism in which lactate-associated PARP1 K654 lactylation enhances DDR activity and contributes to tumor cell survival under cytotoxic stress [31].

Whether lactylation broadly regulates other DDR components remains to be determined. Studies from other cancer models have implicated lactylation of NBS1 and MRE11 in DNA damage sensing and homologous recombination (HR), while XLF- and RAD51-associated lactylation events may influence non-homologous end joining (NHEJ) and HR efficiency, respectively [87,88,89,90,91]. These observations suggest that lactylation may regulate multiple repair processes rather than a single repair factor. However, the relevant modification sites, regulatory enzymes, and therapeutic implications have not yet been established in ESCC.

Lactate availability may also shape lactylation-related responses indirectly through broader metabolic remodeling. In ESCC, studies of circular RNA GOT1 (circGOT1), GLUT1, ALC1, and CCT4 have shown that glycolytic alterations can influence cisplatin sensitivity [66,67,68,69]. Changes in metabolic state, hypoxia adaptation, and redox balance have likewise been associated with radiosensitivity [72,73,74,75]. These findings indicate that metabolic reprogramming may create a cellular environment favorable for lactylation-associated adaptation, although the contribution of specific lactylation events to these effects remains to be defined.

### 4.2. Lactylation-Mediated Regulation of Programmed Cell Death Sensitivity: Adaptation to Ferroptosis and Cuproptosis

#### 4.2.1. Ferroptosis Adaptation

Ferroptosis is an iron-dependent form of regulated cell death driven by lipid peroxidation and controlled by GPX4 and other antioxidant defense pathways [92,93]. Because radiotherapy and several chemotherapeutic agents induce ROS accumulation and oxidative damage, ferroptotic sensitivity has become an important factor influencing tumor responses to treatment [94,95].

In ESCC, multiple studies have implicated ferroptosis defense in treatment adaptation. Radiation-induced lipid peroxidation can be buffered by the nuclear factor erythroid 2-related factor 2 (NRF2)–solute carrier family 7 member 11 (SLC7A11) axis, and increased NRF2/SLC7A11 expression is associated with poorer therapeutic responses [96]. In a cohort of 97 ESCC patients treated with NAC, persistent expression of GPX4 or ferroptosis suppressor protein 1 (FSP1) after treatment was associated with inferior pathological responses, and sustained GPX4 positivity was linked to shorter recurrence-free survival. Functional experiments further showed that GPX4 inhibition increased cisplatin and 5-fluorouracil sensitivity in a subset of ESCC cells [97]. Together, these findings implicate ferroptosis defense in ESCC treatment adaptation and motivate studies of its regulation by lactate and lactylation.

A direct connection between lactate–lactylation signaling and ferroptosis adaptation in ESCC has been demonstrated in studies examining intratumoral microbiota. Lactobacillus-derived D-lactate was reported to induce STAT3 K631 lactylation, promote STAT3 nuclear localization, and increase GPX4 and FTH1 expression, thereby reducing lipid peroxidation and susceptibility to ferroptosis [32]. This work provides an ESCC-specific example in which a microbiota-derived metabolite is linked to a lactylation event and ferroptosis regulation. However, current evidence primarily derives from mechanistic studies and associative clinical analyses, and whether this pathway directly contributes to resistance to radiotherapy, chemotherapy, or immunotherapy remains unresolved.

The biochemical process through which D-lactate contributes to the lactylation substrate pool and enables site-specific modification also requires further clarification. Studies from other malignancies have reported additional connections between lactylation and ferroptosis control, including HDAC1 lactylation and histone lactylation-mediated regulation of the glutamate-cysteine ligase catalytic subunit (GCLC) axis in colorectal cancer [98,99]. These observations extend the mechanistic scope of lactylation-related ferroptosis regulation, but their relevance to ESCC requires disease-specific confirmation.

#### 4.2.2. Cuproptosis Adaptation as an ESCC-Specific Lactylation Mechanism

Cuproptosis provides another example of regulated cell death influenced by lactylation in ESCC. This form of cell death is driven by copper-dependent interactions with lipoylated tricarboxylic acid (TCA) cycle proteins, leading to protein aggregation, depletion of iron–sulfur cluster proteins, and metabolic collapse. Ferredoxin 1 (FDX1) is a key regulator of this process. In ESCC, L-lactate has been reported to induce alanyl-tRNA synthetase 1 (AARS1)-mediated NUDT21 K23 lactylation, whereas histone deacetylase 2 (HDAC2) contributes to removal of this modification. NUDT21 K23 lactylation enhances its interaction with cleavage and polyadenylation specificity factor 6 (CPSF6), promoting formation of the cleavage factor Im (CFIm) complex and extension of the 3′ untranslated region of FDX1. Consequently, FDX1 protein production is reduced, resulting in decreased sensitivity to cuproptosis [33]. The reported synergy between the LDHA inhibitor stiripentol and the copper ionophore elesclomol supports the preclinical therapeutic potential of this pathway [33].

NUDT21 K23 lactylation therefore provides one of the clearest ESCC examples in which a specific lactylation event is linked to regulation of programmed cell death. This mechanism is distinct from the DDR-related mechanisms discussed above. Together with ferroptosis-related findings, these data suggest that lactylation may influence tumor cell survival by altering the activation thresholds of different death pathways under treatment stress. However, ferroptosis and cuproptosis arise from distinct biological contexts and rely on different molecular machinery. Future studies should define their individual relationships with lactylation to determine whether distinct death pathways are regulated through context-specific mechanisms.

### 4.3. The Lactate–Lactylation Axis and Immunotherapy Response in ESCC: From Mechanistic Evidence to Translational Perspectives

Immunotherapy responses differ from tumor-intrinsic adaptations such as enhanced DNA repair or altered cell death sensitivity because they emerge from interactions between malignant cells, immune populations, and the surrounding tumor microenvironment. The lactate–lactylation axis influences this process by reshaping immune states within ESCC. However, its clinical relevance as a determinant of ICI benefit remains to be established. Existing studies have mainly linked lactylation-associated mechanisms to immune phenotypes in ESCC, including impaired immune cell function and establishment of immunosuppressive conditions. Whether these molecular changes directly determine the extent or durability of ICI benefit remains unknown and requires confirmation in prospective clinical cohorts.

#### 4.3.1. Experimental Evidence Linking Lactylation-Associated Mechanisms to ICI Responses

To date, two lactylation-related pathways have been linked to ICI-associated responses in ESCC, although the supporting evidence differs in depth between these pathways. The NIPAL1–HCK–phosphorylated LDHA–H3K18la axis provides a relatively well-characterized example connecting lactate metabolism, lactylation, and immune regulation [36]. In this pathway, NIPAL1 recruits HCK, which promotes LDHA phosphorylation at Y10, enhances glycolytic activity, and increases lactate production. The resulting lactate accumulation facilitates p300-mediated histone H3K18la, linking metabolic changes with epigenetic regulation. Analyses of patient samples have identified associations between this molecular signature and anti-PD-1 responses, while functional studies in MC38 allografts suggest that disrupting this pathway can restore CD8^+^ T-cell activity and improve anti-PD-1 efficacy. Together, these findings associate lactate metabolism and H3K18la with immune therapy response in ESCC, but direct H3K18 residue-level causal testing, ESCC-specific anti-PD-1 efficacy experiments, and prospective validation remain outstanding. HIF-1α K172 lactylation represents another mechanism involved in ICI response regulation [34]. Hypoxia-induced modification of HIF-1α at K172 promotes HIF-1α/hypoxia-inducible factor 1β (HIF-1β) complex formation and enhances glycolytic and immunosuppressive transcriptional programs, including those involving tumor necrosis factor-α (TNF-α) and Notch signaling. Inhibition of hypoxia or interference with this lactylation event increases intratumoral CD8^+^ T-cell infiltration and restores expression of granzyme B, interferon-γ (IFN-γ), and TNF-α. The combination of the HIF-1α inhibitor PX-478 with anti-PD-1 therapy has also shown improved tumor control in preclinical ESCC models [34]. These data suggest that site-specific lactylation may shape ICI responsiveness; however, whether these alterations can serve as predictive biomarkers in patients remains to be determined.

#### 4.3.2. ESCC Evidence Supporting Lactylation-Mediated Immunosuppressive Phenotypes

Studies in ESCC have also demonstrated that lactylation-associated mechanisms may contribute to the formation of an immunosuppressive tumor microenvironment, although their relationship with ICI benefit has not been directly established. Eva-1 homolog A (EVA1A) enhances glycolytic activity and lactate production in ESCC cells, leading to increased histone H4 lysine 12 lactylation (H4K12la) and activation of plasminogen activator urokinase (PLAU) expression. This process is associated with reduced CD8^+^ T-cell activity, whereas EVA1A depletion decreases lactate accumulation and H4K12la levels, resulting in reduced tumor growth and attenuated immune evasion [35]. These findings indicate that lactate accumulation and histone lactylation participate in shaping immunosuppressive states in ESCC. However, whether this lactylation-associated phenotype can be used to predict immunotherapy response or provide a therapeutic opportunity remains unresolved.

#### 4.3.3. Evidence Linking the Lactate Metabolic Microenvironment to Immune Ecosystem Remodeling in ESCC

Beyond the lactylation-related pathways discussed above, alterations in the lactate metabolic microenvironment may influence immune regulation in ESCC. Chromobox protein 6 (CBX6) has been reported to promote C-C motif chemokine ligand 8 (CCL8) secretion and MCT4 expression through SWI/SNF-related matrix-associated actin-dependent regulator of chromatin subfamily D member 1 (SMARCD1)-mediated chromatin remodeling. This metabolic remodeling increases lactate export, reduces CD8^+^ T-cell cytotoxicity, and promotes T-cell exhaustion. CBX6 depletion enhances antitumor immune activity, while tissue microarray analyses have linked CBX6/SMARCD1 expression with immunosuppressive features and unfavorable prognosis [37]. However, this study primarily implicates lactate metabolic remodeling in immune regulation rather than directly demonstrating lactylation-dependent mechanisms. Therefore, these findings should be interpreted as evidence for lactate-associated immune modulation rather than direct evidence of lactylation-dependent regulation. Clinical studies have also reported associations between baseline serum lactate dehydrogenase (LDH) levels, their longitudinal changes, and outcomes in advanced ESCC patients receiving anti-PD-1 therapy [100,101,102]. Circulating LDH, however, reflects multiple biological processes, including tumor burden, tissue damage, hepatic function, and systemic inflammation, and cannot be used as a direct indicator of intratumoral lactate levels or lactylation status.

Taken together, current evidence supports a role for lactylation-associated mechanisms in immune regulation in ESCC, but the strength of support varies substantially among individual mechanisms. HIF-1α K172 lactylation has residue-level causal support in preclinical ICI models. NIPAL1–H3K18la is supported by patient associations and functional studies without direct H3K18 residue-level perturbation. EVA1A–H4K12la mainly supports a role for lactylation in shaping immunosuppressive phenotypes. By contrast, studies focusing on lactate metabolism largely offer indirect evidence for immune microenvironment remodeling and cannot yet be assigned to lactylation-dependent pathways. Further investigation using paired pre- and post-immunotherapy samples, multi-omics approaches, and functional studies will be required to clarify how lactate sources, site-specific lactylation events, and immune cell states interact during treatment. At present, the lactate–lactylation axis is best viewed as an emerging regulatory network connecting metabolic alterations with adaptive immune responses, rather than a universally established mechanism of treatment resistance. Individual lactylation events should therefore be evaluated according to treatment context, causal evidence, and clinically relevant endpoints. The current evidence hierarchy and major limitations are summarized in Table 1 and Table S1.

Evidence quality and bias assessment by tier. To facilitate critical interpretation of the evidence summarized in Table 1 and Table 2, we considered the principal sources of bias and uncertainty at each tier. Tier 1 studies, despite direct residue-level manipulation, remain susceptible to patient-selection bias and limited external validity in single-center cohorts, retrospective association bias when archival tissues are used without treatment-response endpoints, and translational limitations because cell-line or animal models may not fully reproduce the metabolic and immune heterogeneity of human ESCC. Site-specific conclusions may also be influenced by antibody specificity and by non-physiological perturbations, including high exogenous lactate concentrations or broad writer/eraser inhibition. Tier 2 evidence is additionally vulnerable to pleiotropic effects of lactylation regulators, upstream metabolic confounding from glycolytic or LDHA manipulation, and chromatin-association bias, whereby enrichment of a histone lactylation mark does not by itself establish causal regulation by the modified residue. Causal direction may also remain uncertain in patient association studies, and immune findings from syngeneic models may not fully translate to humans. Tier 3 carries the greatest risk of overinterpretation because lactylation dependence may be inferred from lactate-associated biology alone, compounded by cross-cancer or cross-species extrapolation and uncertainty regarding the temporal relationship between lactate accumulation and lactylation changes. These limitations should therefore be considered when balancing mechanistic certainty against translational potential.

## 5. Potential Translational Strategies and Future Validation Directions: From Lactate Metabolic Remodeling to Lactylation Regulation

The lactate–lactylation axis provides a potential framework for developing strategies to counteract therapeutic adaptation in ESCC. Current approaches fall into three categories: reducing lactate production or transport, modulating lactylation-related machinery, and targeting downstream responses associated with specific lactylation events. However, most supporting evidence comes from experimental models, animal studies, or investigations in other malignancies, and clinical validation in ESCC remains scarce. These approaches should therefore be viewed as mechanism-guided candidates rather than established therapeutic options. Future studies are needed to define their biological specificity, safety, and the patient subsets most likely to benefit. Key translational opportunities and unresolved validation issues are summarized in Table 2.

### 5.1. Targeting Lactate Production, Transport, and Microenvironmental Accumulation

Modulating lactate availability represents an upstream approach to counteract the lactate–lactylation axis. LDHA, which converts pyruvate into lactate, is a major determinant of tumor lactate production, whereas lactate dehydrogenase B (LDHB) mainly participates in lactate oxidation and metabolic flexibility. Pharmacological inhibition of LDHA has shown antitumor activity across multiple cancer models, supporting the feasibility of targeting lactate metabolism [103]. In ESCC, LDHA inhibition with stiripentol combined with the copper ionophore elesclomol enhances antitumor effects, suggesting that disruption of lactate metabolism may increase tumor susceptibility to programmed cell death [33]. Similarly, circGOT1 promotes glycolysis, lactate production, and cisplatin tolerance in ESCC, while circGOT1 depletion restores drug sensitivity [66]. These findings indicate that enhanced lactate generation may contribute to treatment adaptation. However, evidence involving sodium dichloroacetate and other metabolic interventions has largely originated from non-ESCC models or nonspecific glycolytic modulation. Such approaches should therefore not be considered direct strategies for reversing lactylation-dependent resistance in ESCC. Whether reducing lactate availability can reshape lactylation patterns and improve treatment outcomes remains to be established.

Lactate transport offers another target for metabolic intervention. Monocarboxylate transporters (MCTs) regulate lactate exchange between tumor cells and their surrounding environment, with MCT4 serving as a major lactate exporter in glycolytic and hypoxic tumors. In ESCC, the CBX6–SMARCD1–MCT4 axis provides disease-specific evidence linking lactate transport remodeling with changes in the tumor metabolic microenvironment [37]. Studies in other cancer models have further shown that targeting MCT4 [solute carrier family 16 member 3 (*SLC16A3*)] can alter immune microenvironmental features and enhance anti-PD-1 responses, whereas inhibition of monocarboxylate transporter 11 (MCT11)–mediated lactate uptake may improve exhausted CD8^+^ T-cell function [104,105,106]. These findings identify lactate transport as a potential interface between tumor metabolism and immune regulation. The MCT1 inhibitor AZD3965 has entered phase I dose-escalation studies in advanced solid tumors and lymphoma, supporting the clinical feasibility of targeting lactate transport [107]. Preclinical studies have also suggested effects on radiosensitivity and immune infiltration [108,109]. Future applications will likely require biomarker-guided selection based on MCT expression, lactate flux, and tumor-specific metabolic characteristics.

Extracellular lactate depletion represents an additional strategy. Lactate oxidase (LOX)-based nanodelivery systems can reduce extracellular lactate levels and enhance antitumor T-cell activity and immunotherapy responses in non-ESCC models [110]. Their relevance to ESCC, however, remains unknown. A disease-specific consideration is the contribution of intratumoral microbial metabolism. Lactobacillus-derived D-lactate has been reported to promote GPX4/FTH1 expression through STAT3 K631 lactylation and enhance ferroptosis resistance in ESCC [32]. This raises the possibility that targeting D-lactate-producing microbial populations or related metabolic pathways could provide another intervention avenue. However, microbiota-directed strategies may affect immune function, epithelial barrier homeostasis, and broader metabolic networks simultaneously. Their therapeutic value therefore requires mechanistic studies that establish causality and define appropriate clinical contexts.

### 5.2. Targeting Lactylation Machinery and Lactylation-Dependent Effector Pathways

Experimental interventions relevant to the lactate–lactylation axis include modulation of lactylation-regulatory machinery and inhibition of downstream effectors. No clinical study has yet directly targeted lactylation in ESCC. These approaches therefore remain at the mechanistic or early preclinical stage.

#### 5.2.1. Targeting the Lactylation Regulatory Machinery

Lactylation dynamics are influenced by the availability of lactyl-group donors, enzymes responsible for modification, and delactylation processes. p300/CREB-binding protein (CBP) functions as an important writer of histone lactylation, and catalytic inhibitors such as A-485 and CPI-1612 have been used as chemical tools to investigate p300-dependent lactylation mechanisms [23,111,112]. However, p300 regulates a broad range of transcriptional and epigenetic programs beyond lactylation [113]. Consequently, phenotypic effects induced by p300 inhibition cannot be attributed solely to reduced lactylation. Future studies should combine site-specific lactylation editing with functional rescue experiments to determine whether individual lactylation events, rather than global suppression of p300 activity, drive changes in therapeutic response.

#### 5.2.2. Targeting Lactylation-Dependent Effector Pathways

Interventions directed at downstream effectors of individual lactylation events may offer greater selectivity than global suppression of lactylation. In ESCC, several site-specific regulatory mechanisms have been directly characterized, including SIRT2-mediated removal of PARP1 K654 lactylation, HDAC2-mediated removal of NUDT21 K23 lactylation, and AARS1-mediated installation of NUDT21 K23 lactylation [31,33]. For the STAT3 K631 lactylation–GPX4/FTH1 axis, targeting STAT3 or GPX4 may offer a route to enhance ferroptosis sensitivity [32]. However, these interventions should not be interpreted as lactylation-specific strategies, because their effects may arise from broader modulation of ferroptosis-related pathways rather than alteration of STAT3 K631 lactylation itself. Defining whether therapeutic responses depend on changes in this specific lactylation event will require direct manipulation of the modification site and appropriate rescue experiments. Beyond lactylation-mediated regulation, studies in other cancer types have shown that additional post-translational mechanisms controlling GPX4 stability, including zinc finger DHHC-type palmitoyltransferase 8 (ZDHHC8)-mediated palmitoylation and ubiquitin-specific protease 8 (USP8)-mediated regulation of GPX4 homeostasis, may also affect ferroptosis and immunotherapy responses [114,115,116,117]. These observations indicate that ferroptosis sensitivity is governed by a broader post-translational regulatory network, within which lactylation represents one potential but not exclusive layer of control.

### 5.3. Translational Opportunities and Candidate Biomarker Frameworks

Current mechanistic evidence supports a biomarker framework for the lactate–lactylation axis that operates across four dimensions: lactate source, lactylation status, downstream biological effects, and clinical outcomes. Potential indicators of lactate availability include circulating lactate, serum LDH, tumor LDHA/MCT4 expression, and intratumoral Lactobacillus-derived D-lactate signatures [38]. However, circulating lactate and LDH are influenced by multiple systemic factors and lack tumor specificity; their interpretation requires integration with tissue-based measurements and treatment context. Lactylation-related biomarkers may include both global lysine lactylation patterns and causally tested site-specific events, such as PARP1 K654, STAT3 K631, NUDT21 K23, and HIF-1α K172 lactylation, together with the lactylation-associated H3K18la signal. These molecular features may be integrated with downstream indicators reflecting DNA damage response activity, programmed cell death states, and immune characteristics [118]. In the immunotherapy setting, standardized assessment of PD-L1, including scoring systems such as combined positive score (CPS), tumor proportion score (TPS), and tumor area positivity (TAP), as well as consistency in sampling location and timing, is essential for improving clinical interpretation [11,12,13]. Translation into clinical applications will require longitudinal and treatment-specific study designs. Samples collected before therapy, during early treatment exposure, and at surgery or disease progression should be linked with treatment information, pathological response, and survival outcomes. PARP1 K654 lactylation may be best evaluated in neoadjuvant chemotherapy or chemoradiotherapy cohorts, whereas the NIPAL1 axis and HIF-1α K172 lactylation require separate assessment in ICI-treated populations. Lactate-related prognostic signatures and circulating biomarkers may serve as exploratory tools, but their clinical utility requires confirmation in independent cohorts [18,38,118].

The purpose of this framework is not simply to increase the number of measurable biomarkers, but to determine whether lactate sources, lactylation states, and downstream functional changes can together explain differences in treatment response among patients. Current human evidence in ESCC is strongest for lactylation-associated adaptation under cytotoxic stress, while its predictive value in immunotherapy remains largely hypothesis-generating. Future studies combining longitudinal clinical cohorts, multi-omics profiling, and functional validation are needed to determine whether the lactate–lactylation axis can support treatment stratification in ESCC.

### 5.4. Safety, Therapeutic Window, and Off-Target Considerations

Although targeting the lactate–lactylation axis is mechanistically attractive, most available interventions act on broadly expressed metabolic or epigenetic regulators and therefore lack lactylation specificity. Their clinical value will depend on whether antitumor activity can be separated from systemic toxicity. LDHA inhibition may reduce tumor lactate production and sensitize ESCC cells to cell death induction, but LDHA is also required for physiological glycolysis, redox balance, and immune-cell metabolism. Stiripentol has clinical safety data from its use as an antiseizure drug [119]. However, the exposure needed to suppress intratumoral lactate or lactylation in ESCC has not been linked to a defined anticancer therapeutic window. The stiripentol–elesclomol combination should therefore remain a preclinical proof of concept rather than a clinically actionable regimen [33].

MCT1 inhibition has reached early clinical testing. AZD3965 has shown pharmacological feasibility in phase I studies, but reversible ocular toxicity, metabolic effects, and compensatory MCT4 expression may restrict its therapeutic window and favor biomarker-guided selection of MCT1-high/MCT4-low tumors [107]. A-485 remains preclinical. In an MC38 model, A-485 at 20 mg kg^−1^ enhanced anti-PD-1 activity. Its efficacy in ESCC, clinical safety profile, and therapeutic window remain unestablished [36]. Moreover, p300/CBP regulates acetylation and broad transcriptional programs in addition to lactylation [111], making both efficacy and toxicity difficult to attribute specifically to H3K18la suppression.

PX-478 enhanced anti-PD-1 activity in preclinical ESCC models at 40 mg kg^−1^ [34], but systemic HIF-1α inhibition may produce hematological and other systemic toxicities because HIF-1α is required for physiological hypoxic responses [120]. Computational and systems-level modeling may further support rational combination-regimen design by informing dose, schedule, and therapeutic-window optimization [121]. Overall, these agents should be considered mechanism-guided candidates. Future development should prioritize biomarker-defined patient selection, tumor-selective delivery, pharmacodynamic monitoring, and, where feasible, interventions directed at validated site-specific lactylation events or immediate downstream effectors.

## 6. Conclusions and Future Perspectives

The lactate–lactylation axis provides a framework for interpreting how metabolic stress translates into adaptive phenotypes in esophageal squamous cell carcinoma (ESCC). Current evidence supports a structured hierarchy rather than a single, uniform mechanism. Site-specific events have been functionally validated: PARP1 K654 lactylation enhances PARylation and DNA damage repair; STAT3 K631 and NUDT21 K23 lactylation alter vulnerability to ferroptosis and cuproptosis; and HIF-1α K172 lactylation provides a preclinical link to hypoxia-associated immune evasion. Together, these studies link metabolic state to adaptive phenotypes through defined lactylation events.

Broader lactylation-associated and metabolic-remodeling findings provide biological context, but do not themselves establish a causal role for a defined lactylation event. Inhibiting LDHA, MCT1, p300/CBP, or HIF-1α can alter lactate-related phenotypes, but none of these interventions is lactylation-specific. Their ESCC therapeutic windows and lactylation-dependent effects remain unresolved. Interpretation is further limited by uncertainty over whether writers and erasers distinguish L- from D-lactate-derived substrates and over acetylation–lactylation competition at shared lysine residues.

Progress will require validated site-specific assays, spatial mapping of lactate sources, and stereochemical characterization of the modification machinery. Longitudinal, treatment-annotated cohorts should test whether lactylation signatures predict response independently of established clinical variables. Priority should be given to prospective validation of ICI-associated mechanisms, including NIPAL1–H3K18la, and to tumor-selective approaches targeting validated lactylation events or their immediate effectors. The lactate–lactylation axis therefore represents a set of testable metabolic–epigenetic hypotheses for improving treatment stratification and precision intervention in ESCC.

## Figures and Tables

**Table 2:** Evidence hierarchy, translational potential and knowledge gaps of the lactate–lactylation axis in ESCC.

Biological Process	Representative Mechanism	Evidence Tier	Evidence Source & Status	Translational Potential	Major Knowledge Gap
DNA damage response	PARP1 K654la → enhanced PARylation → DNA-repair activation	1	Paired ESCC lactylome (*n* = 31) + PARP1 K654R mutagenesis + cisplatin response assays [31]. Direct site-specific mechanism	K654la as candidate pharmacodynamic/stratification marker; PARP1-lactylation-directed interventions require preclinical validation	Prospective paired biopsies; independent validation cohort; incremental predictive value beyond established DDR markers (BRCAness, HRD score)
Ferroptosis resistance	STAT3 K631la → GPX4/FTH1 upregulation → antioxidant defense	1	Multi-regional ESCC microbiome–metabolome–lactylome (27 pts, 102 blocks) + STAT3 K631R validation [32]. Direct source-to-site mechanism	Microbiome/D-lactate profiling for ferroptosis-vulnerability stratification; combination with cytotoxic regimens	L- vs. D-lactate source resolution; longitudinal causality; therapy-specific rescue strategies
Cuproptosis resistance	NUDT21 K23la → FDX1 3′ UTR lengthening → FDX1 protein reduction	1	ESCC tissues + NUDT21 K23R SDM + rescue + tumor models [33]. Direct site-specific mechanism	LDHA inhibition + copper-ionophore (elesclomol) combination; hypothesis-generating, requires clinical validation	Patient selection; systemic copper-toxicity management; dosing optimization; head-to-head validation vs. standard-of-care
Immune checkpoint adaptation	HIF-1α K172la → immunosuppressive transcription	1	155-sample RNA-seq + 12-sample IHC + MS site mapping + K172R SDM + preclinical PX-478 + anti–PD-1 testing [34]. Direct site-specific mechanism	PX-478 (40 mg/kg) + anti–PD-1 synergy; K172la as biomarker for hypoxia-driven immune-cold tumors	Prospective ICB cohorts with HIF-1α inhibitor combination; clinical-grade K172la site-specific assays; validation vs. established hypoxia markers
Immune escape	EVA1A–H4K12la–PLAU → CD8+ T-cell dysfunction	2	ESCC patient samples + EVA1A KD/OE + immune-functional validation [35]. Functional lactylation-associated mechanism; no residue-level causal test or ICB endpoint	H4K12la/PLAU biomarker development; hypothesis-driven ICI combination trials	Direct ICB efficacy testing; prospective ICB cohorts; intervention selectivity and safety
Immune checkpoint adaptation	NIPAL1–HCK–p-LDHA–H3K18la circuit	2	Patient association (60 pts) + immune-competent anti–PD-1 models [36]. Associative evidence	HCK (A419259) or p300 (A-485) inhibition disrupts loop and sensitizes to anti–PD-1; H3K18la as pharmacodynamic marker	Prospective ICB cohorts; comparative marker value vs. PD-L1/TMB; combination safety profiling; clinical-grade H3K18la assays
Lactate microenvironment remodeling	CBX6–SMARCD1–MCT4-mediated lactate efflux → immune remodeling	3	ESCC co-culture/tumor models + microarrays + retrospective ICB cohorts [37]. Lactate-associated remodeling; no lactylation dependence	MCT4-directed pharmacological intervention; serial lactate/LDH monitoring with immune-spatial profiling	Intratumoral lactate quantification methods; paired lactate–lactylation–immune readouts; formal proof of lactylation dependence

Evidence-tier definitions and bias assessment are identical to those in Table 1 (see Table 1 footnote). Arrows (→) denote the reported regulatory sequence and do not necessarily indicate direct interaction.

## Data Availability

No new data were created or analyzed in this study. Data sharing is not applicable to this article.

## Supplementary Materials

The following supporting information can be downloaded at: https://www.mdpi.com/article/10.3390/cancers18182991/s1, Table S1. Verified quantitative and experimental parameters for representative lactate-lactylation studies in ESCC.

## Abbreviations

Clinical and biological terms. CAF, cancer-associated fibroblast; CD8, cluster of differentiation 8; CPS, combined positive score; DDR, DNA damage response; ESCC, esophageal squamous cell carcinoma; FDG-PET, ^18^F-fluorodeoxyglucose positron emission tomography; HR, homologous recombination; HRD, homologous recombination deficiency; ICB, immune checkpoint blockade; ICI, immune checkpoint inhibitor; LNM, lymph node metastasis; NAC, neoadjuvant chemotherapy; nCRT, neoadjuvant chemoradiotherapy; NHEJ, non-homologous end joining; PBMC, peripheral blood mononuclear cell; PD-1, programmed cell death protein 1; PD-L1, programmed death-ligand 1; ROS, reactive oxygen species; TAP, tumor area positivity; TCA, tricarboxylic acid; TMB, tumor mutational burden; TPS, tumor proportion score; TNF-α, tumor necrosis factor α; IFN-γ, interferon γ. Metabolites, modifications, and methods. acetyl-CoA, acetyl-coenzyme A; lactyl-CoA, lactyl-coenzyme A; Kla, lysine lactylation; K(L-la) and K(D-la), L- and D-lactyllysine, respectively; H3K9la, histone H3 lysine 9 lactylation; H3K18la, histone H3 lysine 18 lactylation; H4K12la, histone H4 lysine 12 lactylation; PTM, post-translational modification; PARylation, poly(ADP-ribosyl)ation; LC-MS/MS, liquid chromatography–tandem mass spectrometry; MS/MS, tandem mass spectrometry; MS, mass spectrometry; ChIP, chromatin immunoprecipitation; ChIP-qPCR, ChIP followed by quantitative PCR; co-IP, co-immunoprecipitation; CUT&Tag, cleavage under targets and tagmentation; IHC, immunohistochemistry; IP-WB, immunoprecipitation–Western blot; KD/OE, knockdown or overexpression; PLA, proximity ligation assay; qPCR, quantitative polymerase chain reaction; RNA-seq, RNA sequencing; SDM, site-directed mutagenesis; UTR, untranslated region. Metabolic and regulatory proteins. AARS1, alanyl-tRNA synthetase 1; ACSS2, acyl-CoA synthetase short-chain family member 2; CBP, CREB-binding protein; CDK7, cyclin-dependent kinase 7; DCBLD1, discoidin, CUB and LCCL domain containing 1; DPF2, double PHD fingers 2; GLUT1, glucose transporter 1; GTPSCS, GTP-specific succinyl-CoA synthetase; HDAC, histone deacetylase; HIF-1α/β, hypoxia-inducible factor 1α/β; HK2, hexokinase 2; KAT2A, lysine acetyltransferase 2A; LDH, lactate dehydrogenase; LDHA/LDHB/LDHD, lactate dehydrogenase A/B/D; ldhD, bacterial lactate dehydrogenase D gene; p-LDHA, phosphorylated LDHA; LOX, lactate oxidase; MCT, monocarboxylate transporter; MCT1/MCT4/MCT11, monocarboxylate transporters 1/4/11; SHMT2, serine hydroxymethyltransferase 2; SIRT1/2/3, sirtuin 1/2/3; SLC16A3, solute carrier family 16 member 3; YAP, Yes-associated protein. Mechanism-associated proteins and genes. ALC1, amplified in liver cancer 1; BRCAness, a BRCA-like DNA-repair-deficient phenotype; CBX6, chromobox 6; CCL8, C-C motif chemokine ligand 8; CCT4, chaperonin containing TCP1 subunit 4; CFIm, cleavage factor Im; circGOT1, circular RNA derived from GOT1; GOT1, glutamic-oxaloacetic transaminase 1; CPSF6, cleavage and polyadenylation specificity factor 6; DGCR5, DiGeorge syndrome critical region gene 5; ENO1, enolase 1; EVA1A, Eva-1 homolog A; FDX1, ferredoxin 1; FSP1, ferroptosis suppressor protein 1; FTH1, ferritin heavy chain 1; GCLC, glutamate-cysteine ligase catalytic subunit; GPX4, glutathione peroxidase 4; H2BC9, histone H2B clustered 9; HCK, hematopoietic cell kinase; LAMC2, laminin subunit γ2; MC38, murine colon adenocarcinoma cell line; MRE11, meiotic recombination 11 homolog; NBS1, nibrin; NF-κB, nuclear factor κB; NIPAL1, NIPA-like domain containing 1; NNMT, nicotinamide N-methyltransferase; NRF2, nuclear factor erythroid 2-related factor 2; NUDT21, nudix hydrolase 21; PARP1, poly(ADP-ribose) polymerase 1; PLAU, plasminogen activator urokinase; RAD51, RAD51 recombinase; SLC7A11, solute carrier family 7 member 11; SMARCD1, SWI/SNF-related matrix-associated actin-dependent regulator of chromatin subfamily D member 1; STAT3, signal transducer and activator of transcription 3; TIGAR, TP53-induced glycolysis and apoptosis regulator; USP8, ubiquitin-specific protease 8; XLF, XRCC4-like factor; ZDHHC8, zinc finger DHHC-type palmitoyltransferase 8; WGR, Trp-Gly-Arg domain; HD, helical domain.
