# Supplementary material for: The Lactate–Lactylation Axis as a Metabolic–Epigenetic Framework for Therapeutic Adaptation in Esophageal Squamous Cell Carcinoma
Source: Cancers (Basel). 2026 Sep 16;18(18):2991. doi: 10.3390/cancers18182991 (PMC13604821; doi:10.3390/cancers18182991)
Supplement: Supplementary file 1 [file cancers-18-02991-s001.zip › cancers-4537383-supplementary.pdf]

Table S1. Verified quantitative and experimental parameters for representative lactate-lactylation studies in ESCC.

Reference	Ref. No.	Clinical Material / Cohort	Key Quantitative or Omics Findings	Representative Interventions / Exposure	In Vivo Model	Information Retained in Main Table 1
Peng et al. (2026)	[31]	31 ESCC patients; 62 tumor and adjacent-tissue samples; NAC-treated and untreated groups	8,281 proteins; 1,836 Kla sites; 307 differentially expressed Kla sites	Cisplatin 3 µM for 24 h (sensitivity) or 6 µM for 12 h (mechanistic assay); L-sodium lactate 10 mM for 24 h	No therapeutic animal-intervention experiment reported	PARP1 K654la; Tier 1; site-specific causal validation
Wang et al. (2026)	[32]	27 ESCC patients; 102 multiregional tumor/adjacent-tissue blocks; paired lactate analysis n = 13	Lactobacillus: OS HR 14.09 (95% CI 2.48-80.06; p < 0.01); PFS HR 3.64 (95% CI 1.13-11.73; p < 0.05). Of 994 converged bootstrap models, 96.2% retained HR > 1.	Sodium racemic lactate 20 mM for 24 h; RSL3 100 nM; AZD3965 used, exact concentration not explicitly reported	Eca-109 nude-mouse xenografts; peritumoral L. reuteri twice weekly; i.p. Stattic once daily; n = 6/group; Stattic dose not explicitly reported	STAT3 K631la; Tier 1; source-to-site mechanism
Lin et al. (2025)	[33]	IHC cohort: 251 ESCC cases; TCGA ESCC analysis n = 95	IHC strata: n = 60, 65, 67 and 59; survival association assessed in 251 cases. Other numerical associations are not reproduced here unless explicitly reported in the primary figures.	L-lactate 20 mM; tetrathiomolybdate 1 µM in rescue assays	KYSE30 nude-mouse xenografts; elesclomol 25 mg/kg i.p., three times/week; stiripentol 150 mg/kg i.p., 5 days/week; n = 6/group	NUDT21 K23la; Tier 1; AARS1/HDAC2 regulation
Cong et al. (2026)	[34]	155 paired ESCC RNA-seq samples; 12 clinical samples examined by IF	HIF-1α K172 lactylation identified by MS as a single reported site	PX-478 40 mg/kg/day; TH-302 50 mg/kg/day; anti-PD-1 200 µg/mouse every other day	AKR allografts in immunocompetent C57BL/6 mice; n = 6/group	HIF-1α K172la; Tier 1; ICB-related mechanism
Yuan et al. (2025)	[35]	TCGA: 96 ESCC and 3 normal samples; IHC/immune-correlation cohort n = 24	EVA1A-CD8+ T-cell correlation: Spearman r = -0.83, p < 0.0001 (n = 24); no quantitative lactylome reported	2-Deoxy-D-glucose 4 mM for 24 h	Human PBMC-reconstituted NOG xenografts; n = 6/group	H4K12la-PLAU; Tier 2
Chen et al. (2026)	[36]	52 paired ESCC specimens; PET/CT-IHC cohort n = 30; prospective ICB cohort n = 60	No quantitative lactylome reported. The study reports higher NIPAL1, p-LDHA(Y10), and H3K18la in ICB non-responders.	L-lactate 25 mM; A419259 30 mg/kg once daily; A-485 20 mg/kg once daily; anti-PD-1 10 mg/kg	NIPAL1-overexpressing MC38 allografts in C57BL/6J mice; n = 6/group	NIPAL1-H3K18la; Tier 2
Wang et al. (2025)	[37]	ESCC tissue microarray: 103 patients; anti-PD-1-treated subgroup: 17 patients	No lactylome or residue-specific lactylation analysis	AR-C155858 and AZD0095 used in co-culture; exact concentrations not explicitly reported	mEC25 tumors in nude and C57BL/6 mice; dose not clearly specified in the reported study	CBX6-MCT4 lactate remodeling; Tier 3

All numerical values were checked against accessible primary-study full text, figures, figure legends or Methods. “Not explicitly reported” is retained where the source did not provide a value.
